# Diet, nutrient characteristics and gut microbiome between summer and winter drive adaptive strategies of East China sika deer (*Cervus nippon kopschi*) in the Yangtze River basin

**DOI:** 10.1186/s12866-025-04368-8

**Published:** 2025-10-02

**Authors:** Zhiming Cao, Dandan Wang, Yuanzhen Cui, Fuxing Huang, Yuqin Liu, Jie Dai, Wenguo Wu, Zhijian Dai, Jielei Xie, Xuntao Zhu, Xiaolong Hu, Yongtao Xu

**Affiliations:** 1 Jiangxi Provincial Key Laboratory of Conservation Biology, Jiangxi Agricultural University, Nanchang, 330045 China; 2 Jiangxi Taohongling Sika Deer National Nature Reserve, Pengze, Jiangxi Province 332700 China; 3 School of Public Health, Tianjin University of Traditional Chinese Medicine, Tianjin, 310617 China; 4 College of Animal Science and Technology, Jiangxi Agricultural University, Nanchang, 330045 China

**Keywords:** Adaptation, *Cervus nippon kopschi*, Diet, Nutrition, Gut microbiota

## Abstract

**Background:**

Adaptation of species represents the outcome of interactions between organisms and their environment, as well as a product of natural selection and evolution.

**Method:**

To elucidate how East China sika deer in TNNR respond to seasonal climatic selection pressures in the mid-lower Yangtze River basin, we investigated their seasonal adaptive strategies via analyses of dietary nutrition and the gut microbiome, using high-throughput sequencing of the trnL P6-loop of chloroplast and 16S rRNA.

**Results:**

In summer, sika deer consumed 174 plant species belonging to 183 genera and 107 families, exhibiting pronounced dietary generalization. Conversely, in winter, they fed on 130 species from 173 genera and 90 families, characterized by dietary specialization. The nutritional composition and availability of plants differed between the two seasons, driven by seasonal changes, which led to corresponding adjustments in foraging strategies. Notably, sika deer maintained a stable balance in nutrient intake across seasons. and industrialization of sika deer breeding in eastern China, whereas α-diversity was higher in winter. Microbiota in both seasons exhibited distinct correlations with consumed plant species and nutrients, but their microbial functions were predominantly enriched in metabolic processes. This pattern indicates that sika deer can flexibly reshape the structural and interaction networks of gut microbiota to enhance adaptive capacity to seasonal shifts. Overall, we demonstrated seasonal dynamics and provided new insights into understanding the diet diversity and nutrition components associated with gut microbiota in the adaptation of sika deer. These results will further facilitate genetic resource conservation, habitat improvement, food plant breeding, wild rescue, and industrialization of sika deer breeding in eastern China.

**Supplementary Information:**

The online version contains supplementary material available at 10.1186/s12866-025-04368-8.

## Introduction

Species adaptation is conducive to revealing evolutionary drivers in shaping biodiversity and expounding complex relationships among genotypes, physiology, and environments [1, 2]. In their natural habitats, animals often encounter multifaceted selection pressures, including seasonal variations, food shortages, and interspecies competition. The capacity to modify survival strategies in response to environmental changes is essential for species to ensure the survival of individuals and the entire population [3, 4]. Animals adapt to changing environments by adjusting foraging strategies and gut microbiota composition to enhance adaptive coordination within their habitats [5, 6].

In wildlife adaptation, obtaining nutritious food is crucial. It sustains individual health by fueling metabolic and immune functions. A key aspect of wildlife adaptation to the environment is the ability to acquire nutritionally adequate food to maintain individual health and population reproduction [7]. Factors such as food type, nutritional composition, palatability, and digestibility influence wildlife foraging strategies [8]. The temporal and spatial heterogeneity in plant resource distribution, driven by climate and plant phenology, causes fluctuations in quantity and nutritional quality, often leading to drives seasonal variations in herbivores’ foraging strategies. For example, *Cervus elaphus alashanicus* and *Rusa unicolor* exhibit marked seasonal variations in consumed plant species [9, 10]. Nutritional ecology, a branch of functional ecology focusing on nutritional linkages formed during feeding, investigates organisms’ response strategies to variations in food quantity and nutritional quality [11]. Animal nutritional strategies prioritize different nutrient components based on factors such as body size, physiological status, and energetic demands [12].

The gut microbiota encompasses diverse microbial communities that reside within the digestive systems of animals. These microbial communities have undergone a process of synergistic coevolution, developing intricate mechanisms over extended periods of coexistence with their hosts [13]. The intestinal tract is sterile before birth, but bacteria enter the host via air or food postnatally, gradually colonizing the gut under the selective pressures of the immune system and intestinal environment (i.e., pH and oxygen levels) to form a stable community comparable to the adult microbiome [14]. Gut microbiota mediates critical host functions such as digestion and absorption [15], immune regulation [16], pathogen defense [17], and vitamin synthesis [18]. Within the limited intestinal space, different microbes interact through mutualism and competition to maintain homeostasis [19]. Gut microbiota composition is shaped by multifaceted factors, including host diet [20], genetics [21], age [22], and living environment [23], with food serving as a direct determinant by providing energy for both hosts and microbes while sculpting the microbial habitat [24].

The sika deer (*Cervus nippon*), a member of the Cervidae family within the order Cetartiodactyla, is indigenous to the East Asian monsoon region [25]. It is a first-class national key protected species in China [26]. Anthropogenic activities and climate dynamics have driven the dramatic contraction of their habitats, leading to local extinctions across numerous historical ranges since the Holocene. By the 1940s, only three sika deer subspecies survived in China: *C. n. sichuanicus*, *C. n. mantchuricus*, *and C. n. kopschi* [27]; these remaining subspecies became crucial focal points for conservation efforts. The East China sika deer (*C. n. kopschi*) is confined to specific regions, namely areas in Pengze County, Jiangxi Province (with the largest population), Jixi and She Counties in Anhui Province, and Lin’an district in Zhejiang Province. This subspecies confronts multiple challenges, including low population growth rates, habitat fragmentation, restricted gene flow, and reduced biomass of food plants due to vegetation succession [28]. Prior studies regarding East China sika deer mainly involved dietary patterns [29], activity rhythms [30], environmental adaptation [31] and population genetics [32]. However, these previous studies mainly focused on single-aspect investigations, and a comprehensive understanding of the sika deer’s adaptation mechanisms, especially considering the interaction between diet, nutrition, and gut microbiota, is still lacking.

To clarify how East China sika deer adapt to the climatic selection pressures during summer and winter in the middle and lower reaches of the Yangtze River, this study employed trnL and 16S rRNA sequencing, coupled with the analysis of plant nutritional components to investigate the adaptation mechanisms involving dietary nutrition, as well as the composition and function of the gut microbiota. The findings are expected to enhance germplasm resource conservation, habitat modification, ex-situ conservation practices, and promote the successful reintroduction of the East China sika deer.

## Methods and materials

### Study region and sampling

Taohongling national nature reserve of sika deer (hereafter, TNNR) is situated on the southern bank of the Yangtze River basin. Spanning the coordinates of 29°42′−29°53′N and 116°32′−116°43′E, the TNNR was established in 2001 to protect sika deer and natural habitats [33]. The TNNR features low mountains and hilly topography, and the altitudes range from 100 to 500 m. Its flora comprises 1,034 seed plant species across 529 genera and 139 families, with vegetation dominated by Poaceae-rich herbaceous communities and Rhododendron-dominated shrublands that provide critical forage for herbivores [34, 35]. Situated within the transitional area between the mid-subtropical and warm-temperate zones, TNNR is subject to a warm and humid monsoon climate marked by well-defined seasons. Its summers are typically hot and parched, frequently beset by droughts, and during winters, there is a notable decrease in plant productivity [36]. These complex climatic and edaphic conditions pose significant adaptive challenges to native wildlife, including sika deer [36].

**Fig. 1:**
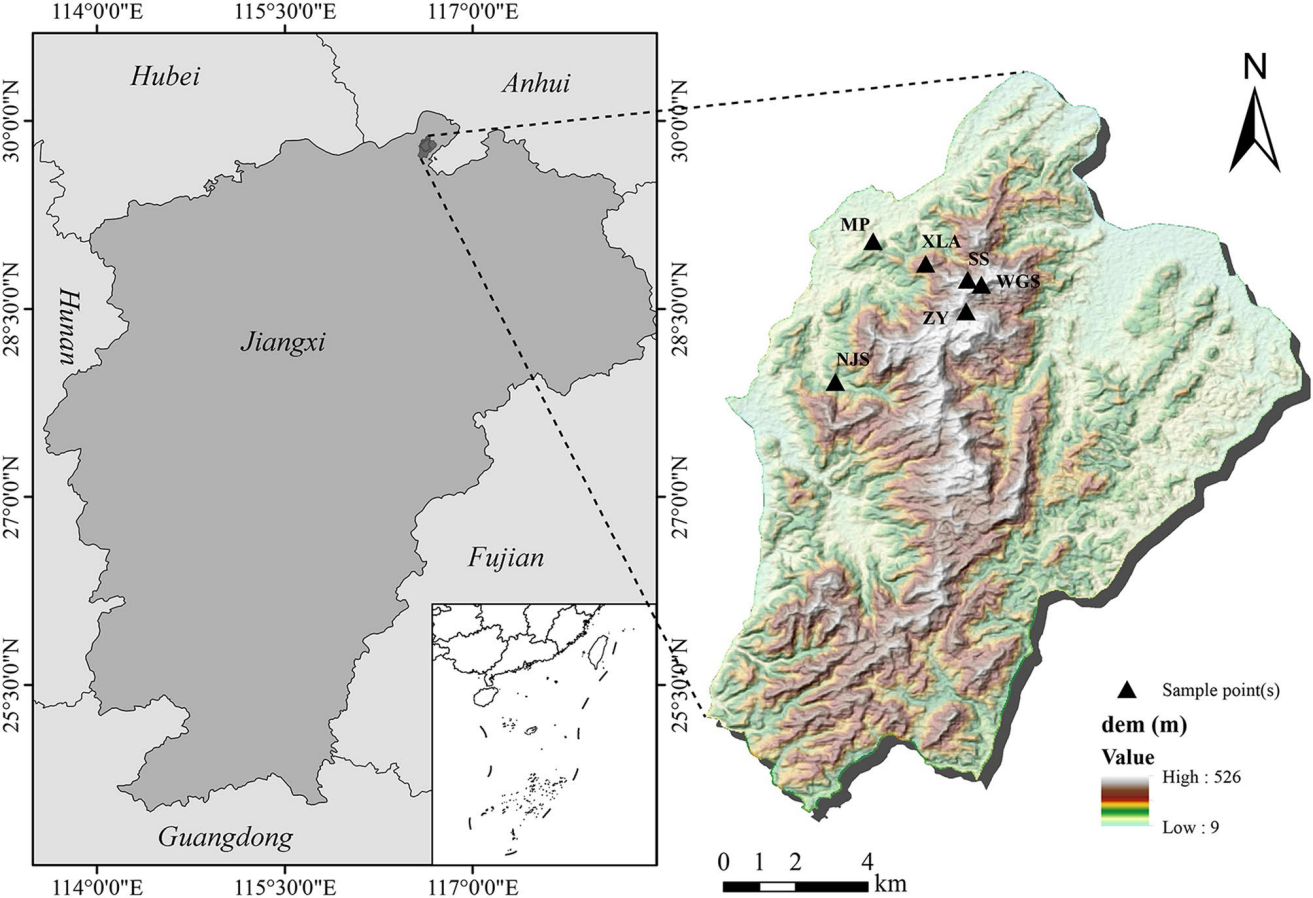
Six feaces sampling sites of sika deer including MP (Nursery bases), NJS (NieJiaShan), XLS (XianLingAn), ZY (Bamboo garden), SS (Fir forests) and WGS (WuGuiShi) in Taohongling Nature Reserve

In this study, fecal samples were collected from six fixed locations in the TNNR, where the sika deer population frequently engaged in daily activities such as drinking water, Licking salt and feeding from June 16th to 20th, 2020 (summer) and from December 8th to 10th, 2020 (winter) (Fig. 1). Based on whether the feces were grayish-brown and peanut in shape, it can be identified whether it comes from sika deer [37]. Fresh fecal samples were collected using sterile, sealed bags. To avoid or minimize sampling redundancy from the same individual, feces exhibiting different morphological characteristics (e.g., size, shape, color) were preferentially selected. In cases where multiple fecal samples were found nearby (30–50 m), only one sample was collected. All samples were immediately placed into insulated containers with ice packs for transport and stored at -80 °C. Altogether, 60 fecal samples were obtained, 30 of which were sampled in summer and 30 in winter.

### DNA extraction, PCR and high-throughput sequencing

The surface of fecal samples was scraped to extract the host DNA and identify the species using 16S ribosomal RNA forward and reverse primers (F: 5′-GAGAAGACCCTATGGAGC-3′ and R: 5′-ATAGAAACCGACCTGGAT-3′). Randomly, five fecal pellets of sika deer were selected from every sample and combined so as to merge one composite sample to decrease the errors. The summer and winter sample groups were numbered SLu001-030 and WLu001-030, respectively. Plant DNA was extracted according to the instructions of the DNA extraction kit (TianGen, Beijing), and microbial flora DNA was extracted according to the instructions of the QIAamp DNA fecal kit (Qiagen, Germany), including cell lysis, protein removal, and DNA purification (Figure S1). Universal trnL metabarcoding derived from chloroplast was g (5’-GGGCAATCCTGAGCCAA-3’) and h (5’-CCATTGAGTCTCTGCACCTATC-3’) [38]. The 16S rRNA gene primers 799F (5’-AACMGGATTAGATACCCKG-3’) and 1193R (5’-ACGTCATCCCCACCTTCC-3’) were utilized to amplify the V5-V7 target region [39]. PCR was carried out on foraging plants and fecal microbiota according to the optimal annealing temperature, and we detected the amplified products through 1.0% agarose gel electrophoresis. Then, Shanghai Personal Biotechnology Co, Ltd. performed the sequencing.

The high-throughput sequencing data were preserved in FASTQ format. The diet sequencing data underwent quality control, denoising, merging, and de-replication processes, which were performed using the Vsearch clustering method. Subsequently, the high-quality sequences were clustered at a 97% similarity level, and representative sequences of operational taxonomic units (OTUs) were generated. Annotation was conducted using a local nt or nr database. The BROCC algorithm along with blastn or blastx was employed to align the sequences with nucleotide sequences from the nt or nr database [3]. The threshold was set as an e-value less than 1e-5 [19, 40]. The most closely related species were compared, and a species-level classification annotation was carried out for each taxonomic unit. OTU sequences that could not be classified at the species level were labeled “Unclassified” [37]. Based on genus-level abundance data, Levin’s niche breadth index was calculated using the *spaa* package in R software (version 4.4.3).

For the analysis of fecal microbiota sequencing data, the Divisive Amplicon Denoising Algorithm 2 approach was implemented [41]. Sequences with 100% similarity were defined as Amplicon Sequence Variants (ASVs). Species annotation was executed using the “classify-sklearn” function within QIIME2’s (Quantitative Insights Into Microbial Ecology version 2) Naive Bayesian classifier framework. A confidence threshold of 0.7 was set for annotation, as described by Bokulich et al. [42]. The Greengenes database (Release13.8; http://greengenes.secondgenome.com/) served as the reference for annotating each ASV sequence at the species level [43]. Based on the annotation outcomes, histograms and heat maps were generated to visualize the distribution of bacterial communities in each sample at the phylum and genus taxonomic levels.

### Nutritional component analysis

Based on the DNA barcode sequencing results, plant taxa with a genus-level relative abundance greater than 1% were selected for further analysis. As sika deer typically forage on tender plant shoots, the browsed plant tips often exhibit clean-cut fracture marks, the identification of foraged species was refined by combining DNA-based results with fresh browsing traces observed in areas of high sika deer activity. Plant species were further identified based on morphological characteristics, with reference to iPlant [44]. For each genus, one widely distributed species showing frequent browsing evidence was selected as a representative. Plant samples were collected during the same phenological period from 2020 to 2021, targeting young shoots. Sample individuals were collected from more than three distinct sites for each species. All plant materials were preserved, transported on dry ice, and stored at −20 °C. Then the samples were minced, oven-dried at 60℃ for 48h, ground into powder using a ball mill, passed through a 1 mm sieve, and stored in sealed bags for subsequent analysis. Nutritional components of food were determined by referring to Li et al. [45] and Rothman et al. [46]. The nutrients of each food item, including available crude protein (CP), soluble sugar (SS), crude fat (CF), starch (ST), neutral detergent fibre (NDF), acid detergent lignin (ADL), acid detergent fibre (ADF), ash, total non-structural carbohydrates (TNC), total structural carbohydrates (TSC), gross energy (GE), and relative feed value (RFV), were analyzed.

To clarify the comprehensive nutritional status of various plants, the maximum values of CP, CF, SS, ST, TNC and TSC, as well as the minimum values of lignin and ash content of the food plants of sika deer in summer and winter, were respectively selected as the reference sequences for the nutritional indicators of the food plants of sika deer in summer and winter. The grey correlation coefficient formula from existing research was adopted [47]. The analysis was conducted by comparing each nutritional index of summer and winter forage plants against the reference sequences. The weighted correlation degrees derived from these analyses were used as final scores, with calculations predominantly performed using the *DT* package in R software (version 4.4.3). This study primarily analyzed macronutrient intake and availability in sika deer across summer and winter. Individual macronutrient intake was calculated as the sum of nutrient contents in each forage plant multiplied by the plant’s proportional contribution to the diet. The differences in the nutritional intake of sika deer between summer and winter were statistically analyzed using One-way ANOVA. The results were visualized using the *ggplot2*, *ggsignif*, and *cowplot* packages in R software (version 4.4.3). The macronutrient balance of sika deer in summer and winter is visualized through the right-angle mixed triangle (RMT) model, which can graphically display the three-component macronutrient mixture [48].

### Data processing

Network analysis was conducted using the sparse inverse covariance estimation with ecological association inference (SPIEC-EASI) method [49]. R software (version 4.4.3) and Gephi were used to visualise the data. Linear Discriminant Analysis Effect Size (LEfSe) was carried out to uncover the significant rankings of abundant modules in the sika deer’s gut microbiota. A size-effect threshold of 4.0 on the logarithmic linear discriminant analysis (LDA) score was applied for discriminative functional biomarkers. Moreover, Alpha diversity indices from rarefied samples, such as observed species, Chao1, Simpson, Shannon, Pielou’s evenness, and Good’s coverage, were computed by QIIME2 using community richness and diversity indices. Permutational multivariate analysis of variance (PERMANOVA) was used to contrast the difference in alpha indices. To measure similarities among groups, principal coordinate analysis (PCoA) and Non-metric multidimensional scaling (NMDS) based on Bray-Curtis distance (to obtain corrected *P-values* and 95% confidence intervals) were performed using the *vegan* package in R software. The Spearman test was used to examine the correlation between the food and the gut microbial composition in each season [50]. Functional profiling of ASVs in each group was performed using PICRUSt2 (Phylogenetic Investigation of Communities by Reconstruction of Unobserved States 2), employing default settings and referencing the Kyoto Encyclopedia of Genes and Genomes (KEGG) database [51].

## Results

### Forages composition and nutrients analysis

**Fig. 2:**
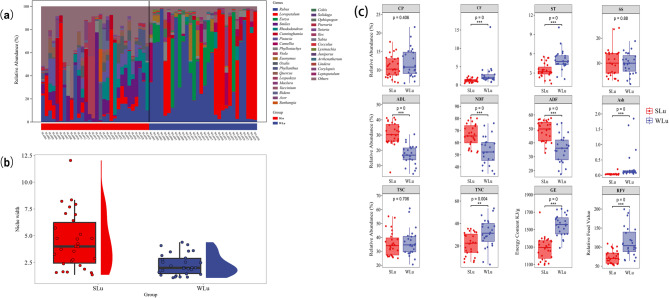
Comparative analyses of diet and nutrition in summer and winter (**a**) The 20 most abundant forage plants at the genus level (**b**) The Levin’s ecological niche width of sika deer at the genus level (**c**) Significant test of plant nutrients component

The trnL sequencing unveiled that sika deer consumed 174 foraging plants from 183 genera and 107 families during summer, with the three most abundant genera being *Smilax* (12.14%), *Rubus* (10.72%), and *Loropetalum* (10.14%). By contrast, the winter diet comprised 130 species from 173 genera and 90 families, dominated by *Rubus* (36.08%), *Loropetalum* (26.24%), and *Eurya* (13.31%) (Fig. 2a). In winter, the combined relative abundance of the top three plant genera consumed by sika deer accounted for 75%, indicating a dietary shift toward specialization. In contrast, summer diets included a broader range of plant genera, with the most abundant genus, *Smilax*, comprising only 12.14% of the total, suggesting no dominant food source and a more generalized foraging strategy. Niche breadth reflects the overall range of resources utilized by a species; a higher niche breadth index indicates a more generalized diet, whereas a lower index suggests dietary specialization. This study calculated Levin’s niche breadth index at the genus level for both seasons. The index was substantially higher in summer (4.48 ± 2.62) than in winter (2.28 ± 0.98), further supporting a contraction of dietary niche breadth in the colder season (Fig. 2b).

Based on sequencing results, 27 summer and 24 winter food plant species were selected for nutrient analysis. Comparative analysis revealed significant seasonal variations in nutrient profiles (Fig. 2c). Winter foraging plants exhibited higher concentrations of crude fat (summer: 1.27 ± 0.52% vs. winter: 2.78 ± 2.94%), starch (3.42 ± 1.11% vs. 5.02 ± 1.61%), ash (0.04 ± 0.03% vs. 0.29 ± 0.48%), total non-structural carbohydrates (21.43 ± 9.94% vs. 31.39 ± 12.73%), gross energy (1,284.62 ± 135.89 vs. 1,552.05 ± 121.43 kJ/g), and relative feed value (73.64 ± 17.19 vs. 115.97 ± 38.64) (Student’s t-test, *P*-value < 0.05). Conversely, summer plants contained higher levels of lignin (30.82 ± 7.72% vs. 17.08 ± 6.48%), neutral detergent fiber (54.28 ± 11.32% vs. 67.08 ± 9.08%), and acid detergent fiber (36.69 ± 10.11% vs. 49.07 ± 7.19%) (*P*-value < 0.05). No significant differences were found in crude protein (10.94 ± 2.89% vs. 11.98 ± 3.90%, *P*-value > 0.05), soluble sugars (10.43 ± 5.64% vs. 9.94 ± 4.31%, *P*-value > 0.05), or total structural carbohydrates (35.46 ± 8.72% vs. 36.18 ± 9.57%, *P*-value > 0.05) (Table S1, 2). A grey relational analysis based on macronutrient content assessed the comprehensive nutritional profile of plant species consumed by sika deer. The analysis revealed that *Pistacia chinensis* ranked highest among summer forage species, while *Eurya japonica* had the highest score in winter (Table S3).

### Comparison of nutrient intake and strategic models

Comparative analysis of macronutrient intake demonstrated that the winter mean values of the sika deer for crude protein (summer: 7.91 ± 2.59% vs. winter: 9.94 ± 0.83%), crude fat (0.94 ± 0.33% vs. 1.49 ± 0.31%), starch (2.52 ± 0.76% vs. 4.28 ± 0.58%), soluble sugars (9.22 ± 3.13% vs. 11.62 ± 2.23%), total non-structural carbohydrates (TNC) (18.14 ± 5.61% vs. 32.43 ± 5.39%), total structural carbohydrates (TSC) (25.07 ± 7.30% vs. 34.22 ± 2.68%) and gross energy (GE) (1,437.13 ± 71.38 kJ/g vs. 965.42 ± 218.69 kJ/g) were significantly higher than those in summer (all the *P*-value < 0.05).

**Fig. 3:**
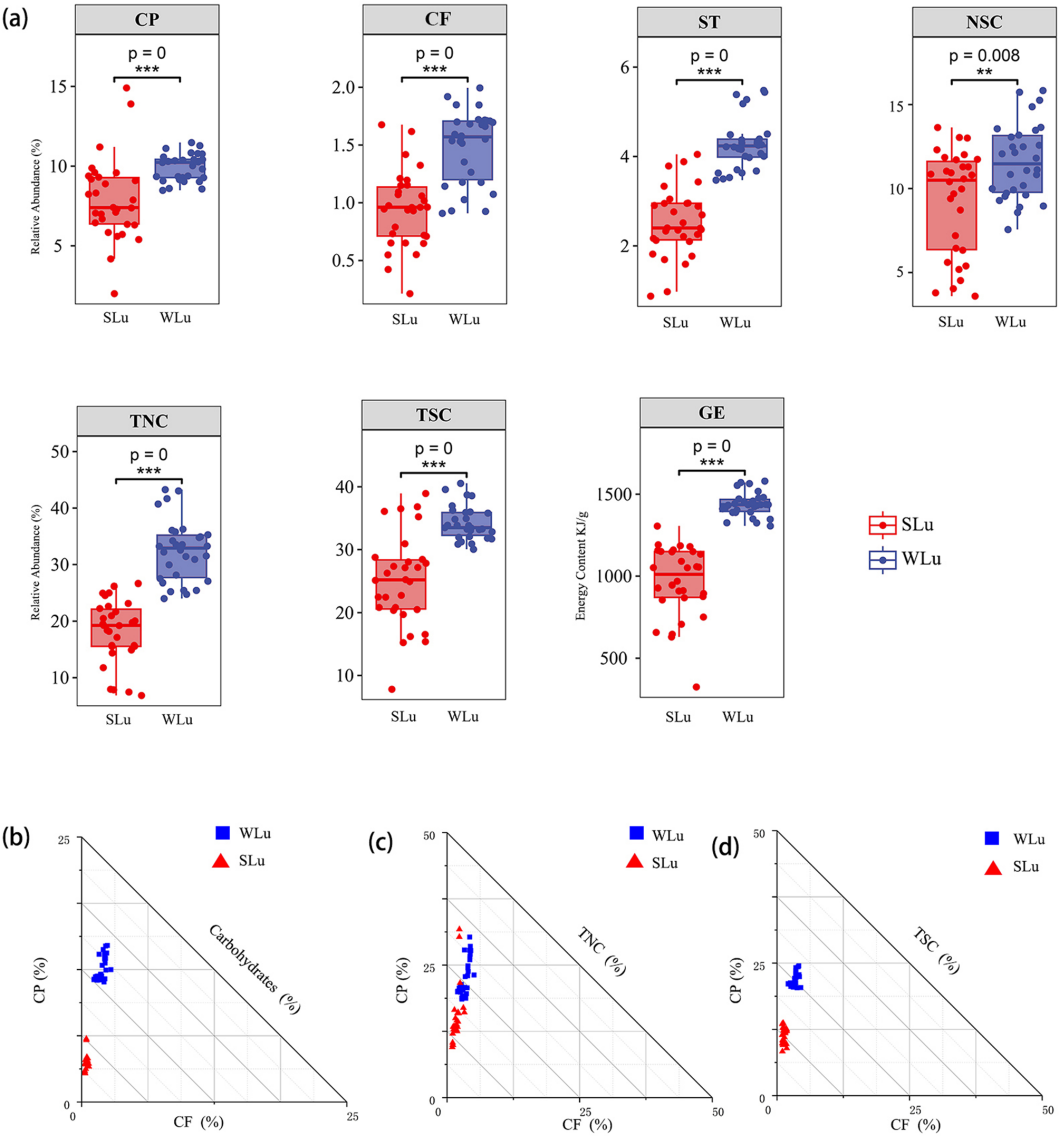
Nutritional intake and balance strategies of sika deer in summer and winter (**a**) Box plot of macronutrient intake strategy. Three-dimensional diagram of the straight triangle model of crude protein, crude fat and carbohydrates (**b**) total non-structural carbohydrates (**c**) and structural carbohydrates (**d)**

The RMT model maps three macronutrients into a three-dimensional coordinate space, representing their relative proportions and enabling a visual interpretation of animal nutritional requirements. This framework was applied to construct seasonal macronutrient intake models for sika deer (Fig. 3b, d), where each axis corresponds to a specific macronutrient. Each point in the model denotes the proportional contribution of carbohydrates, proteins, and lipids to the total macronutrient intake. Model outputs revealed discrete clustering of summer and winter individuals, underscoring divergent intake strategies for the three macronutrients. Across all combinations of species and sites, the ratios of carbohydrates and proteins were more variable than those of lipids. As a result, dietary compositions clustered more closely along the vectors that represented dietary lipid concentrations.

### Gut microbiota

**Fig. 4:**
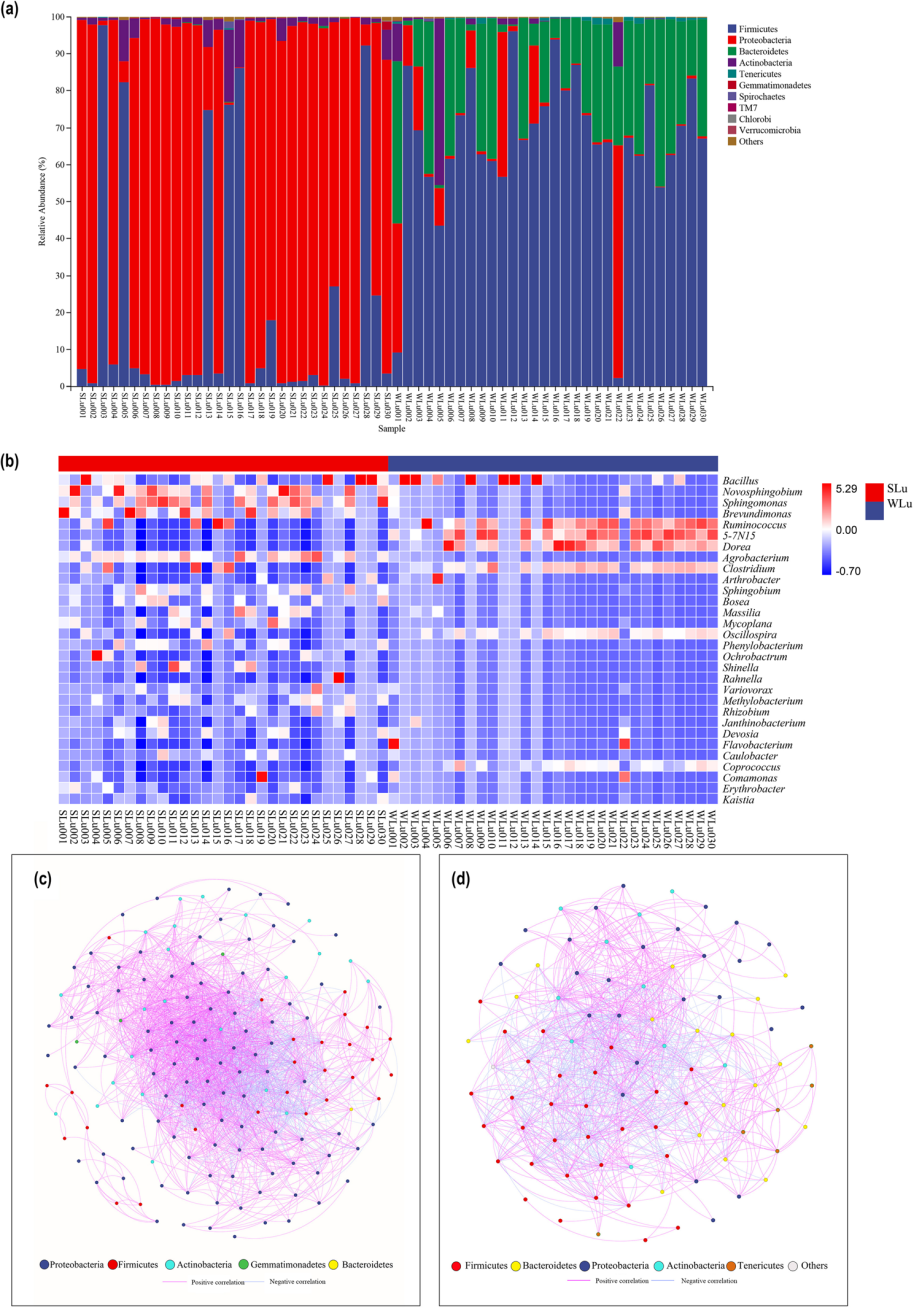
Analyses of gut microflora composition and associated network (**a**) Relative abundance of gut microflora at the phylum level of sika deer in summer and winter (**b**) Abundance heat map of gut microbiota at the genus level in summer and winter.The genus-level gut microflora association network of sika deer in summer (**c**) and winter (**d)**

A total of 20 phyla, 42 classes, 77 orders, 148 families, 317 genera, and 290 species were identified in the gut microbiota across seasons. Summer samples comprised 16 phyla, 33 classes, 62 orders, 119 families, and 243 genera, while winter samples contained 15 phyla, 30 classes, 56 orders, 111 families, and 218 genera. At the phylum level (Fig. 4a), the five most abundant phyla in summer were Proteobacteria (75.12%), Firmicutes (21.03%), Actinobacteria (3.24%), Gemmatimonadetes (0.12%), and Bacteroidetes (0.08%), respectively. In winter, the dominant phyla shifted to Firmicutes (66.44%), Bacteroidetes (22.82%), Proteobacteria (7.39%), Actinobacteria (2.54%), and Tenericutes (0.51%). At the genus level (Fig. 4b), summer microbiota was dominated by *Enterococcus* (10.25%), *Novosphingobium* (5.52%), *Sphingomonas* (5.36%), *Brevundimonas* (4.64%), and *Bacillus* (3.46%). Winter communities were dominated by *Bacillus* (13.72%), *Ruminococcus* (3.80%), *Dorea* (3.29%), *Pseudomonas* (2.33%), and *Clostridium* (1.99%).

To understand the microbial community interactions of different hosts, a SPIEC-EASI co-occurrence network at the genus level was constructed. Overall, the gut microbial networks differed significantly among the control, SLu (Fig. 4c) and WLu group (Fig. 4d). The network of the SLu group contained more nodes and links and was larger than that in the WLu group. The average degree and average clustering coefficients in the SLu group were both larger than those in the WLu group (Table S4). Genus-level co-occurrence network analyses revealed that summer microbiota networks exhibited higher node numbers, connection counts, and average degrees than winter networks, indicating greater complexity and putative resilience.

### Differences and diversity analysis of gut microbiota

**Fig. 5:**
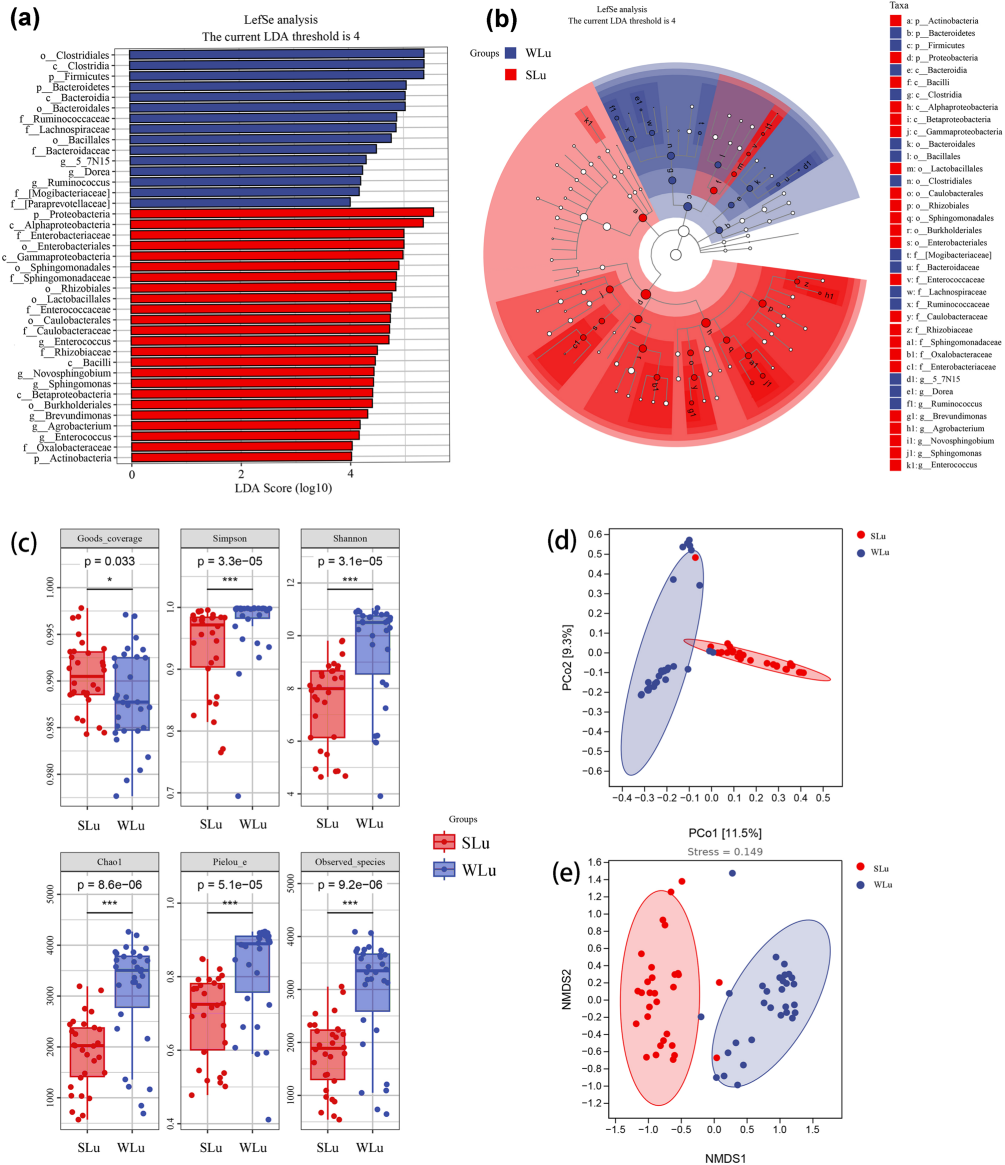
LEfSe analysis (**a** and **b**), Alpha diversity based on Simpson, Shannon, Chao 1, Pielou_e, and Oberserved species indexes (**c**), PCoA analysis (**d**), NMDS analysis (**e**) of the intestinal microflora of sika deer in two season groups

We used LEfSe to identify significant differences in gut microbiota composition between summer and winter groups. In the summer group, one phylum (Proteobacteria), four classes (Alphaproteobacteria, Gammaproteobacteria, Bacilli, Betaproteobacteria), six orders (Enterobacteriales, Sphingomonadales, Rhizobiales, Caulobacterales, Lactobacillales, Burkholderiales), six families (Enterobacteriaceae, Sphingomonadaceae, Caulobacteraceae, Enterococcaceae, Rhizobiaceae, Oxalobacteraceae), and five genera (*Enterococcus*, *Novosphingobium*, *Sphingomonas*, *Brevundimonas*, *Agrobacterium*) were significantly enriched. In the winter group, enriched taxa included two phyla (Firmicutes, Bacteroidetes), two classes (Clostridia, Bacteroidia), three orders (Clostridiales, Bacteroidales, Bacillales), six families (Ruminococcaceae, Lachnospiraceae, Bacteroidaceae, Mogibacteriaceae, Paraprevotellaceae, Clostridiaceae), and four genera (Unnamed genus 5_7N15, *Ruminococcus*, *Dorea*, *Clostridium*) (Fig. 5a, b).

The Good’s coverage index for both seasons surpassed 98.8%, demonstrating that the majority of microbial taxa in the samples were successfully annotated and the results reliably reflect real-world conditions. Notably, the winter gut microbiota demonstrated significantly higher alpha diversity indices—including Simpson, Shannon, Chao1, Pielou_e, and Observed_species—compared to those in summer (Wilcoxon rank-sum test, *P*-value < 0.001; Fig. 5c). PCoA (Fig. 5d) and NMDS (Fig. 5e) analyses based on Bray-Curtis distance matrices were employed to compare gut microbiota across seasons. PCoA revealed distinct clustering of summer and winter samples, while NMDS confirmed significant seasonal differentiation in microbiota composition with a stress value of 0.149, indicating reliable ordination. Both analyses demonstrated pronounced disparities in gut microbial communities between summer and winter.

### Functional prediction and diet-microbiota interaction

**Fig. 6:**
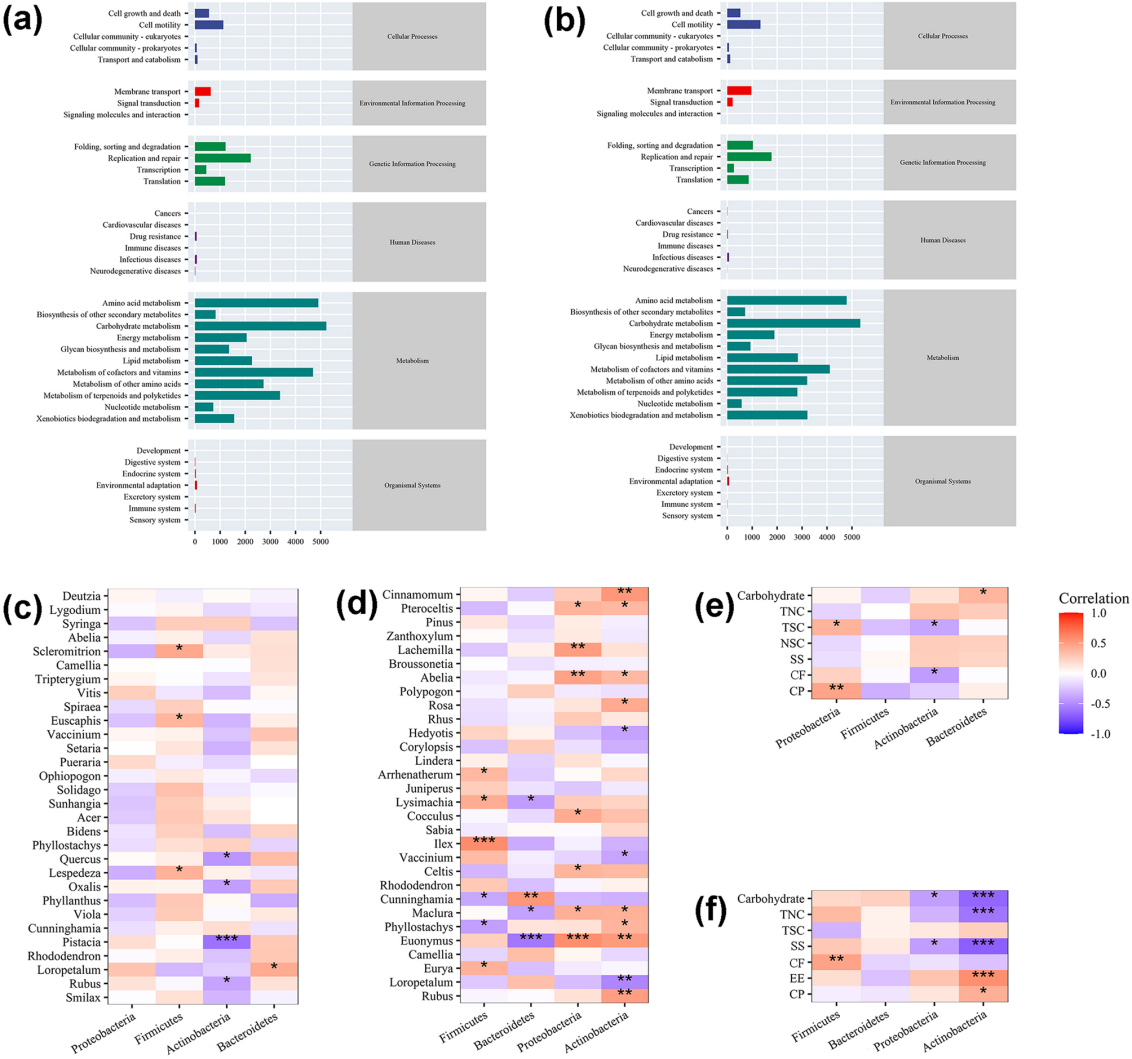
Abundance of KEGG metabolic pathway in intestinal flora of sika deer in summer (**a**) and winter (**b**); Correlation between intestinal flora at the phyla level and ingested plants (**c**) and nutritional components (**e**) in summer, and Spearman correlation between intestinal flora at the phyla level in winter and ingested plants (**d**) and nutritional components (**f**) in winter

To characterize the Functional profiles of summer and winter gut microbiota in sika deer, PICRUSt Functional prediction was applied to 16S rRNA gene sequencing data. By leveraging 16S rRNA taxonomic profiles and performing comparative analysis against the KEGG metabolic database, we found that gut microbial communities in both seasons were predominantly enriched in metabolic functions at the first hierarchical level of KEGG orthology (KO), followed by genetic information processing, environmental information processing, cellular processes, human diseases, and biological systems (Fig. 6a, b). Within the metabolic function category at the second hierarchical level, the top three most abundant gene functional groups across both seasons were *carbohydrate metabolism*, *amino acid metabolism*, and *cofactor/vitamin metabolism*. Notably, summer microbiomes exhibited *other amino acid metabolism* as the fourth function, while winter microbiomes were distinguished by xenobiotic biodegradation and metabolism as the fourth functional category.

Spearman correlation analysis was performed to evaluate associations between gut microbiota (phylum-level) and food plants (genus-level) or ingested nutrients. In summer, Proteobacteria exhibited significant positive correlations with two nutrients; Firmicutes showed positive correlations with two plant genera; Actinobacteria demonstrated significant negative correlations with four plant genera and two nutrient categories; and Bacteroidetes had a positive correlation with one plant genus. In winter, Firmicutes displayed positive correlations with four plant genera and one nutrient category, along with negative correlations with two plant genera; Bacteroidetes was positively correlated with one plant genus and negatively correlated with three plant genera; Proteobacteria showed positive correlations with seven plant genera but a negative correlation with one nutrient category; and Actinobacteria revealed positive correlations with eight plant genera and two nutrient categories, and negative correlations with two plant genera and two nutrient categories.

## Discussion

### Seasonal food composition and nutritional dynamics

Significant seasonal variations occur in the nutritional compositions of foraging plants, such as crude fat and carbohydrate fractions. These seasonal variations in nutritional compositions have a profound impact on the survival and reproduction of sika deer. For example, in winter, the higher fat content in foraging plants provides more energy for sika deer to resist the cold and maintain their basic metabolic needs. Plants adapt to seasonal shifts in temperature, precipitation, and light, which are accompanied by periodic morphological and physiological adjustments [52]. Cellular constituents (e.g., sugars, proteins, fats) generally reach peak levels during the growing season and decrease during winter dormancy, while cell wall components (e.g., lignin, cellulose) exhibit an inverse pattern, underscoring the profound seasonal influences on plant nutrient profiles [53].

Among the plant species consumed by sika deer, *Pistacia chinensis* exhibited the highest overall nutritional value in summer, while *Eurya japonica* ranked highest in winter. However, several of the most frequently consumed species in both seasons contained insufficient levels of certain key nutrients, resulting in lower composite nutritional scores. These findings suggest that the foraging preferences of sika deer may not be solely driven by overall nutritional quality. In addition to these findings, notably, the plant species most frequently consumed by sika deer in summer exhibited relatively high soluble sugar levels. Previous studies have shown that soluble sugars are a key glucose source for rumen microbial protein synthesis in ruminants and are considered a significant determinant of dietary selection in many deer species [12]. This suggests that, under conditions of summer food abundance, sika deer may prefer to forage for plants with higher soluble sugar content. In addition, plants have evolved secondary metabolites such as terpenoids and phenolics to deter herbivory by modulating feeding behavior and intake [54]. Therefore, future studies should explore the relationship between various secondary metabolites and the dietary composition of sika deer to elucidate their adaptive strategies.

In the TNNR, sika deer adopt a foraging strategy characterized by dietary specialization in winter and dietary generalization in summer. In complex wild environments, herbivores frequently encounter spatiotemporal heterogeneity in food resources, driven by fluctuating ecological factors such as temperature, precipitation, and vegetation phenology. Adjusting diet composition and intake in response to phenology-induced resource scarcity represents a common adaptive strategy among ungulates [29]. For instance, plateau pikas (*Ochotona curzoniae*) on the Gannan Grassland consume diverse plant taxa during the growing season but rely on a restricted set of species during the dormant season [4]. Studies in Zhejiang’s Qingliangfeng Nature Reserve demonstrate that sika deer exhibit broad herbivory on forbs and grasses in summer/autumn, shifting to shrub-dominated diets in winter/spring [37]. Winter temperature declines in TNNR, where shrubland is the dominant vegetation type, exacerbate limitations in available plant species and tissues [33, 36]. Evergreen shrubs (*Rubus*, *Loropetalum*, *Eurya*) are widely distributed and serve as primary winter food sources, whereas deciduous species (*Cudrania*, *Celtis*, *Euonymus*) occur patchily in sunlit, sheltered microhabitats. The Optimal Foraging Theory predicts that animals prioritize energy acquisition with minimal foraging effort, explaining why reserve deer concentrate on highly available plant taxa in winter to meet basal metabolic demands [55]. Therefore, with reduced food availability in winter, sika deer in the reserve appear to adopt a foraging strategy that prioritizes highly accessible plant species to meet the energetic demands necessary for survival.

Given the above, native evergreen shrubs such as *Eurya japonica*, *Rosa multiflora*, and *Ilex cornuta* demonstrate relatively high nutritional value and are also commonly used in landscaping with well-established cultivation protocols [56]. It is recommended that the reserve conduct controlled feeding experiments to assess the foraging preferences of sika deer. Based on the results, and after evaluating the availability and ecological risks of the preferred plant species, these palatable species can be cultivated in abandoned farmland near areas of high sika deer activity to help ensure adequate nutritional resources during winter.

### Nutrient intake and balance strategies

Animals employ foraging strategies dependent on environmental resource availability to maintain dynamic nutritional balance and maximize energy acquisition, thus shaping species-specific nutritional tactics [57]. In this study, while protein content in summer and winter food plants did not differ significantly, winter protein intake was notably higher than summer intake. Protein, a digestible and absorbable nutrient, is critical for ungulate survival, growth, and reproduction. Cervids may employ strategic protein intake regulation: for example, *Dama dama* targets specific protein thresholds rather than maximizing intake [58], while *Alces alces* balance protein and non-protein nutrients [59].

Carbohydrates, comprising three-quarters of plant dry weight, are categorized into TSC and TNC. TSC requires fermentation by gut microbiota for host absorption, whereas TNC (including soluble sugars) is directly utilizable by ungulates. Fat, which supplies 13% of an animal’s energy requirements, yields 2.25 times more energy per gram than carbohydrates or proteins [60]. As a primary energy source for ungulates, fat is critical for moose in Inner Mongolia’s Hanma National Nature Reserve, which increase crude fat and carbohydrate intake in winter to mitigate cold stress and food scarcity [61]. Consistently, sika deer in this study exhibited higher winter intake of fat, total carbohydrates, and gross energy, supporting thermoregulation and elevated foraging activity during harsh seasons.

The nutritional balance hypothesis posits that animals modify feeding behavior to maintain nutrient homeostasis, favoring nutritionally balanced or complementary food types and adjusting daily intake patterns [12]. Sika deer exhibited distinct seasonal nutritional strategies: crude fat demonstrated the most stable relative contribution across seasons, whereas carbohydrate and protein intakes fluctuated significantly. Ruminants actively regulate nutrient intake to sustain rumen microbiota functionality: *Alces alces* prioritize total non-structural carbohydrate (TNC) balance [62], and *Capreolus pygargus* in northeastern China adjust plant consumption to maintain macronutrient equilibrium [63].

### Gut microbiota composition and functions prediction

In winter, the top four bacterial phyla (by relative abundance) in the sika deer gut microbiota were Firmicutes, Bacteroidetes, Proteobacteria, and Actinobacteria—structurally similar to the gut microbiomes of other ungulates, including *Cervus elaphus* [6], *Cervus eldii hainanus* [64], *Moschus berezovskii* [65], and *Elaphurus davidianus* [66]. Firmicutes, the primary cellulolytic bacteria in herbivores, degrade plant fiber into volatile fatty acids (VFAs) to facilitate host energy utilization [6]. Bacteroidetes aid in the digestion of proteins and carbohydrates, thereby enhancing nutrient absorption [67]. These two phyla form the foundation of herbivore gut microbiomes, with their abundance ratio strongly correlated with host energy efficiency [68]. Proteobacteria, ubiquitously present in ungulate guts, play critical roles in maintaining intestinal anaerobic conditions and microbial homeostasis [69].

Studies show that mammalian gut microbial community assembly is regulated by multiple factors, including diet, host genetics, and social behavior [70]. In the wild, seasonal fluctuations in food resources frequently drive alterations in gut microbiota composition. For instance, moose in Hanma Nature Reserve exhibit increased Firmicutes abundance in winter to optimize energy extraction from nutritionally restricted diets [61]. Li et al. observed that seasonal nutritional variations induced gut microbiota shifts in Tianxing gibbons (*Hoolock tianxing*) [71], a pattern also documented in *Ochotona curzoniae* [72], *Theropithecus gelada* [73], and *Bison bison* [74]. Our study reinforces that seasonal changes in food resources drove gut microbiota restructuring in sika deer. This study demonstrates that seasonal shifts in food resources drive the restructuring of the gut microbial community in sika deer.

However, functional predictions of the gut microbiota using PICRUSt2 revealed that microbial functions in both seasons were predominantly associated with metabolic processes, with carbohydrate metabolism as the most abundant functional category. As the primary energy source for ungulates, carbohydrates—particularly cellulose and hemicellulose—rely heavily on gut microbial fermentation, which explains the dominance of metabolic functions [75, 76]. Analogous findings in Rhinopithecus demonstrate that seasonal diet shifts alter microbiota composition while preserving core digestive and metabolic functions [77]. PICRUSt2 has been widely used to predict the functional composition of microbial communities [51]. Related studies have shown that the Spearman correlation between PICRUSt2-predicted KO abundances and metagenomic sequencing KO abundances ranges from 0.79 to 0.88 [51]. However, the relatively short length of the 16S rRNA gene (V5–V7 region) may reduce the number of detectable taxa, lower phylogenetic resolution, and lead to the loss of genetic information necessary to distinguish bacterial taxa associated with host species and geographic origin, thereby limiting the accuracy of functional predictions [78].

### Gut microbiota diversity and co-occurrence networks

According to the insurance hypothesis, ecosystems with higher species diversity tend to exhibit greater stability and higher levels of ecological functioning because many species provide greater guarantees that some will maintain functioning even if others fail [79–81]. In this study, sika deer’s gut microbiota exhibited higher diversity in winter despite reduced food resource availability. Previous research has suggested that increased microbial diversity enhances the stability of the gut microbial ecosystem. For example, *Pseudois nayaur* in Sanjiangyuan National Park, which inhabits rocky cliff areas with limited plant resources, harbors significantly more diverse gut microbiotas than *Ovis aries* and *Pantholops hodgsonii* in plains, thereby enhancing nutrient utilization capacity [19]. Furthermore, diverse bacterial communities in the gut contribute unique digestive enzymes, enhancing food fermentation efficiency [82]. Li et al. found that *Ochotona curzoniae* at high altitudes, facing sparser food resources, recruit more diverse gut bacteria from the environment to improve digestive functionality [72].

Within the constrained intestinal environment, microbial taxa form complex interaction networks via neutralism, commensalism, amensalism, competition, and predation while competing for resources [83, 84]. Summer gut microbiota in sika deer exhibited higher network complexity and stronger inter-taxa correlations, likely linked to their more diverse diet. Huang observed complex, highly connected gut microbiota networks in wild *Cervus eldi* during peak food availability, a pattern consistent with our findings [63]. During the green season, plateau pikas consuming toxic plants alongside a diverse diet rely on complex microbial networks for food detoxification [85]. To adapt to summer dietary diversity, sika deer may optimize microbial interactions, forming intricate co-occurrence and metabolic networks to enhance digestive flexibility.

### Interactions between seasonal diet-nutrition and gut microbiota

Under the constraints of limited nutrients and metabolic substrates provided by the host to the gut microbiota, different microbial taxa exhibit divergent competitive abilities for nutritional resources. Consequently, gut microbiota often exhibit distinct positive/negative associations with dietary components [86]. Our correlation analysis between gut microbiota and foraged plant abundance revealed that dominant microbial taxa displayed distinct positive/negative associations with specific plant species, likely driven by inherent variations in plant chemical compositions. Qian et al. demonstrated that due to heterogeneity in nutrient and chemical profiles, bacterial communities colonizing different food fractions in the rumen of *Cervus elaphus yarkandensis* diverged post-digestion [87]. Zhang et al. further confirmed that gut microbiota composition in *Ovis ammon polii* is closely linked to consumed plant species, with hosts actively modulating microbial structure through food selection to adapt to seasonal nutritional demands and environmental stresses [88].

Additionally, correlation analysis between gut microbiota and macronutrient intake revealed significant seasonal variations in microbe-nutrient associations, potentially linked to seasonal differences in nutrient uptake efficiencies. For example, *Theropithecus gelada* on the Ethiopian Plateau increase the abundance of cellulolytic and fermenting bacteria (i.e., Ruminococcaceae, Lachnospiraceae) during the rainy season, when consuming cellulose-rich grasses, whereas during the dry season, they enrich starch-decomposing and methanogenic taxa (e.g., Succinivibrionaceae, Streptococcaceae) while feeding on starchy, lignified underground roots [73].

## Conclusions

Plant nutrient composition and availability in the TNNR exhibit pronounced seasonal variation. *Pistacia chinensis* had the highest comprehensive nutritional score in summer, while *Eurya japonica* dominated winter diets. Fluctuating food resources drove sika deer to adjust foraging strategies with dietary generalization in summer (broad intake of plant species) and specialization in winter (concentration on evergreen shrubs), with both seasons maintaining balanced nutrient intake. Summer and winter gut microbiota exhibited distinct compositional differences, with seasonal variations in correlations between microbial taxa and dietary plants/nutrients. Despite structural shifts, microbial functions remained concentrated in metabolic pathways, particularly carbohydrate metabolism, a key process for digesting plant fiber. This functional consistency underscores the microbiota’s role in sustaining energy acquisition across seasons. Winter gut microbiota showed higher alpha diversity, likely enhancing nutrient utilization from limited food resources via increased digestive enzyme diversity. In contrast, summer microbiota formed more complex co-occurrence networks, optimizing microbial interactions to process diverse dietary inputs. These findings highlight the capacity of sika deer to dynamically adjust microbiota structure and inter-taxa relationships, enabling resilience to seasonal environmental fluctuations. Future studies could focus on the long-term effects of climate change on the diet-nutrition-gut microbiota axis of sika deer and explore more effective conservation strategies based on these findings.

## Supplementary Information

Supplementary Material 1.

## Data Availability

The raw sequence data have been deposited in the NCBI Short Read Archive as BioProject (Accession numbers: PRJNA1122487 and PRJNA1122507), and the available link is http://www.ncbi.nlm.nih.gov/sra.

## Abbreviations

TNNR: Taohongling national nature reserve of sika deer
OTUs: Operational taxonomic units
ASVs: Amplicon sequence variants
QIIME2: Quantitative insights into microbial ecology version 2
LDA: Linear discriminant analysis
PICRUSt2: Phylogenetic investigation of communities by reconstruction of unobserved states 2
PERMANOVA: Permutational multivariate analysis of variance
PCoA: Principal coordinate analysis
NMDS: Non-metric multidimensional scaling
KEGG: Kyoto encyclopedia of genes and genomes
CP: Crude protein
SS: Soluble sugar
CF: Crude fat
ST: Starch
NDF: Neutral detergent fibre
ADL: Acid detergent lignin
ADF: Acid detergent fibre
TNC: Total non-structural carbohydrates
TSC: Total structural carbohydrates
GE: Gross energy
RFV: Relative feed value
RMT: Right-angle mixed triangle
SPIEC-EASI: Sparse inverse covariance estimation with ecological association inference
