# Supplementary material for: Diet, nutrient characteristics and gut microbiome between summer and winter drive adaptive strategies of East China sika deer (Cervus nippon kopschi) in the Yangtze River basin
Source: BMC Microbiol. 2025 Oct 2;25:626. doi: 10.1186/s12866-025-04368-8 (PMC12492541; doi:10.1186/s12866-025-04368-8)
Supplement: Supplementary file 1 — Supplementary Material 1. [file 12866_2025_4368_MOESM1_ESM.docx]

**Supplementary file**


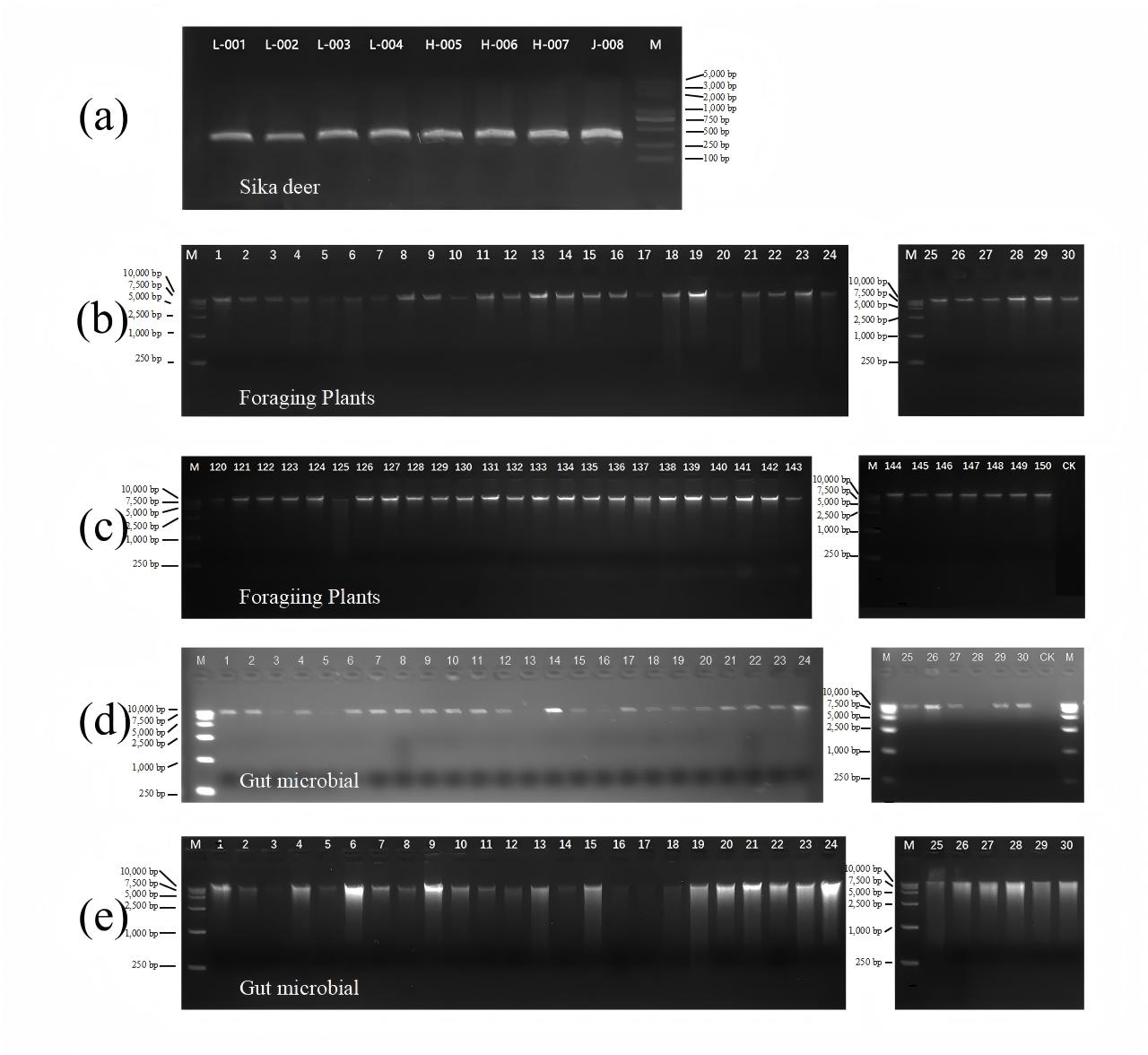


Figure S1 Gels image of DNA extractions and PCR products. (a) displays the PCR product of host (sika deer) DNA with an expected fragment size of approximately 300 bp. (b) and (c) represent the plant DNA extracted from fecal samples in summer and winter, respectively. (d) and (e) show the gut microbial DNA of sika deer extracted in summer and winter, respectively.


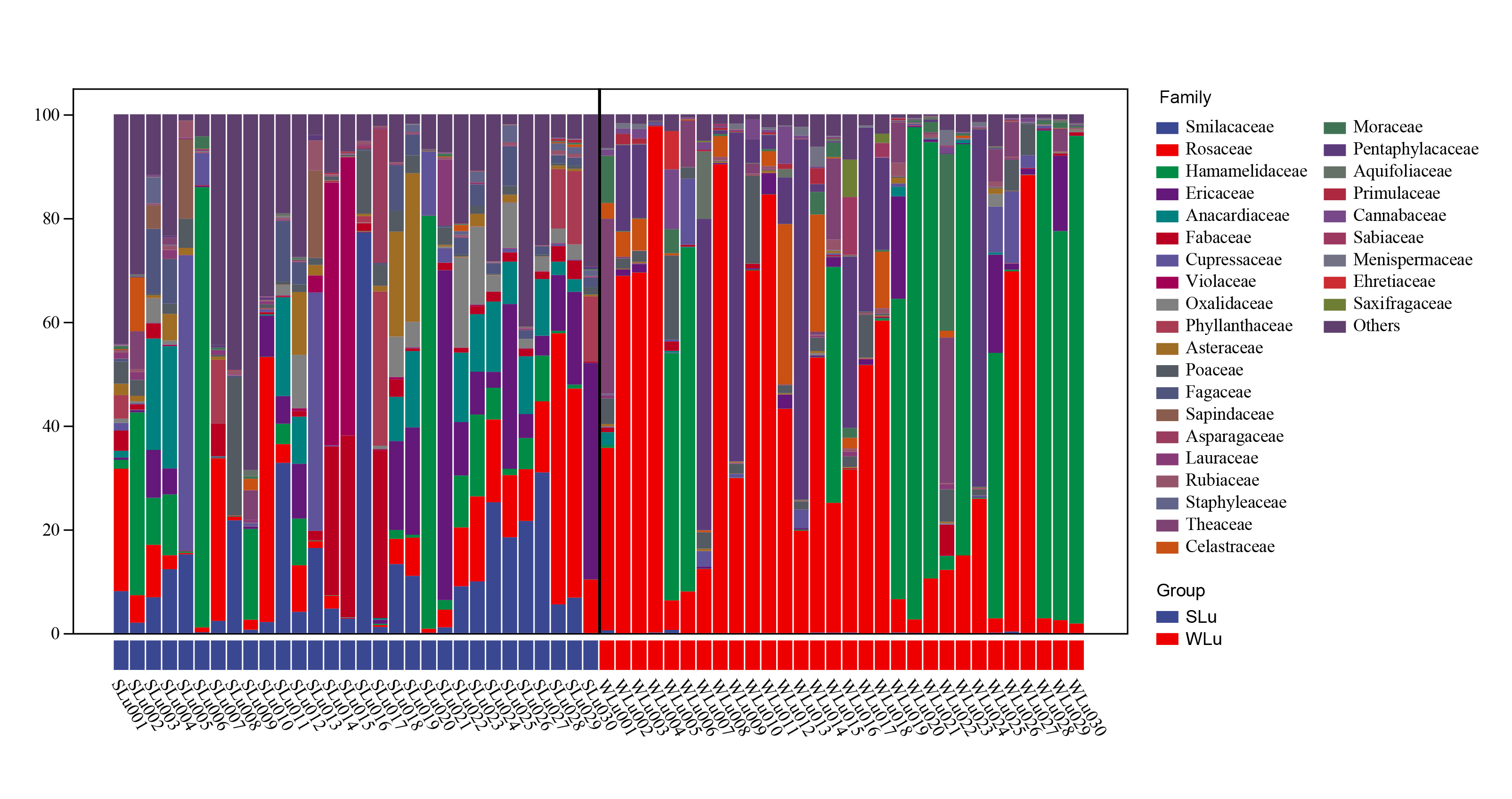


Figure S2 The 20 most abundant forage plants at the family level

(a)


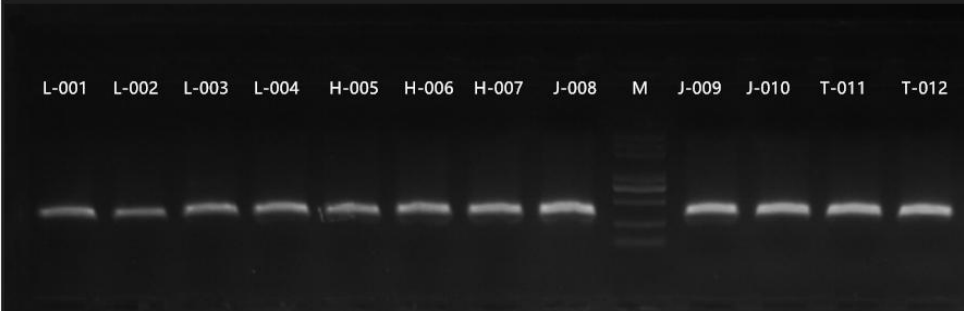


(b)


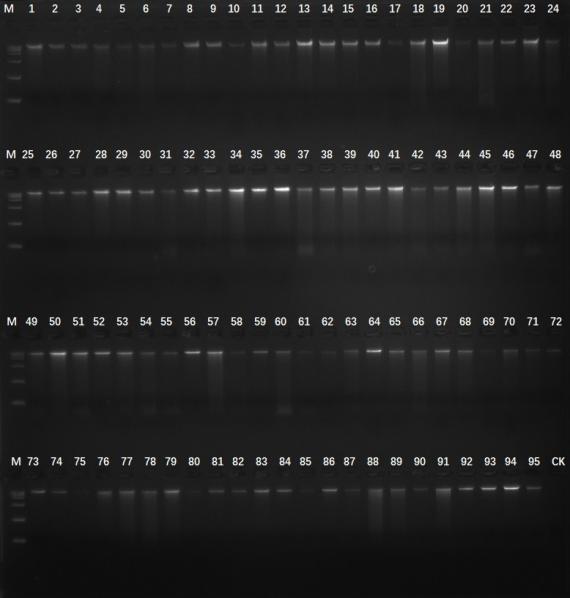


(c)


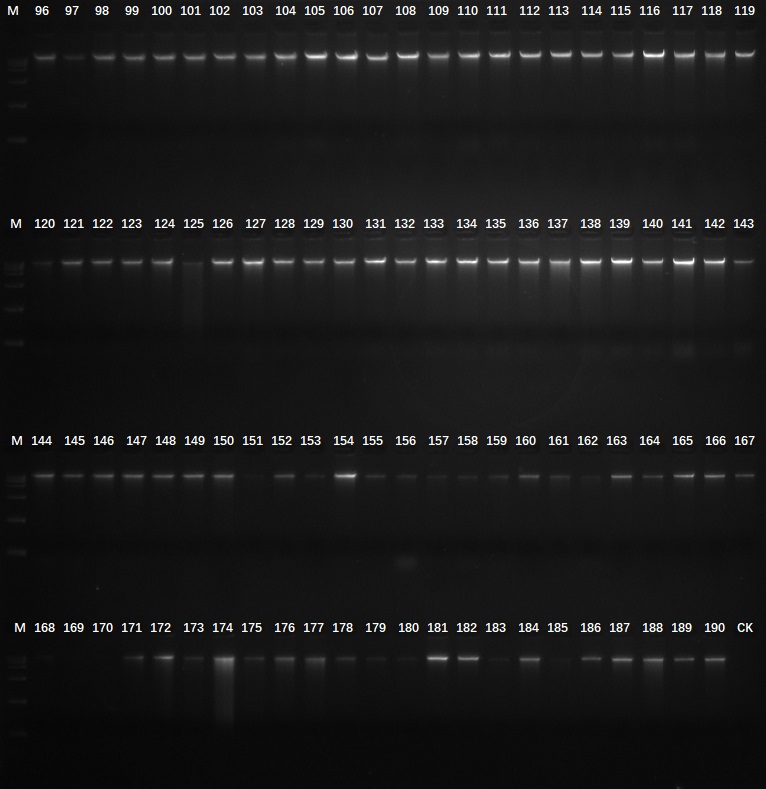


(d)


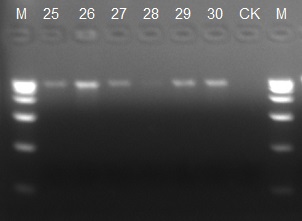


(e)


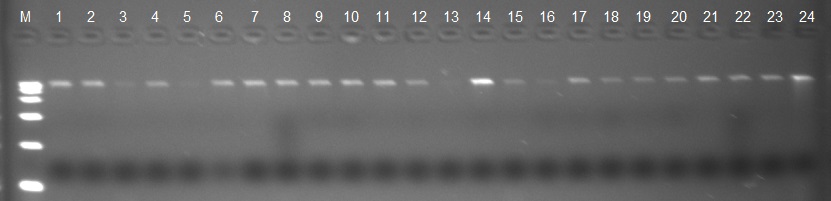


(f)


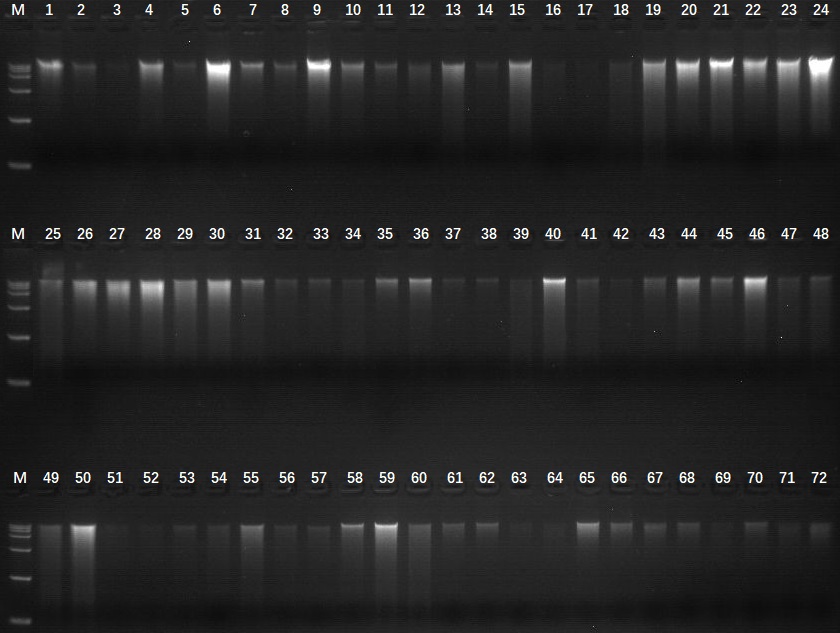


Figure S3 The full uncropped Gels images: (a) Gel electrophoresis results of molecular identification for sika deer based on the mitochondrial 16S rRNA gene (samples L-001 to L-004, ~300 bp), (b)Total genomic DNA of foraging plants from fecal samples in summer (No.1 - No.30, M: Marker, CK: negative control), (c) Total genomic DNA of foraging plants from fecal samples in winter (No.121-150), (d) Total genomic DNA of gut microbiota from fecal samples in summer (No.1-24), (e) Total genomic DNA of gut microbiota from fecal samples in summer (No.25-30), (f) Total genomic DNA of gut microbiota from fecal samples in winter (No.1-30)

.

**Table S1** Main nutrients composition of diet plants for sika deer in summer

| **Species** | **Genus** | **CP** | **EE** | **ST** | **SS** | **ADL** | **NDF** | **ADF** | **Ash** | **TSC** | **TNC** | **GE** | **RFV** |
| --- | --- | --- | --- | --- | --- | --- | --- | --- | --- | --- | --- | --- | --- |
| *Smilax china* | *Smilax* | 10.06 | 1.50 | 2.64 | 14.23 | 38.20 | 65.40 | 52.80 | 0.04 | 65.40 | 23.00 | 1813.19 | 67.95 |
| *Rubus alceifolius* | *Rubus* | 10.15 | 0.80 | 2.99 | 15.23 | 27.40 | 53.70 | 41.30 | 0.04 | 53.60 | 35.31 | 1796.38 | 98.27 |
| *[Loropetalum chinense](http://www.iplant.cn/info/Loropetalum chinense)* | *Loropetalum* | 6.63 | 0.60 | 3.51 | 14.09 | 37.40 | 63.10 | 47.20 | 0.04 | 63.09 | 29.63 | 1770.61 | 76.85 |
| *Rhododendron simsii* | *Rhododendron* | 7.37 | 0.90 | 3.10 | 5.99 | 39.20 | 77.70 | 54.40 | 0.03 | 77.60 | 14.00 | 1780.75 | 55.70 |
| *Pistacia chinensis* | *Pistacia* | 10.26 | 2.40 | 2.55 | 23.60 | 25.70 | 51.90 | 35.90 | 0.03 | 51.90 | 35.41 | 1834.81 | 109.22 |
| *Cunninghamia lanceolata* | *Cunninghamia* | 7.96 | 2.00 | 5.04 | 13.71 | 35.50 | 80.70 | 56.10 | 0.04 | 80.60 | 9.30 | 1809.04 | 52.10 |
| *Viola philippica* | *Viola* | 17.64 | 1.20 | 3.10 | 3.76 | 26.00 | 73.80 | 44.80 | 0.04 | 73.70 | 7.32 | 1853.95 | 68.07 |
| *Oxalis corniculata* | *Oxalis* | 11.06 | 1.10 | 5.37 | 5.19 | 41.20 | 71.90 | 56.70 | 0.05 | 71.90 | 15.89 | 1810.55 | 57.87 |
| *Phyllanthus urinaria* | *Phyllanthus* | 13.19 | 1.20 | 3.19 | 3.05 | 26.40 | 52.90 | 39.90 | 0.03 | 52.90 | 32.68 | 1826.96 | 101.67 |
| *Quercus fabri* | *Quercus* | 11.36 | 0.60 | 3.16 | 13.84 | 32.80 | 64.80 | 48.90 | 0.04 | 64.80 | 23.20 | 1801.47 | 72.94 |
| *Lespedeza pilosa* | *Lespedeza* | 14.80 | 0.70 | 1.89 | 3.79 | 40.10 | 76.00 | 56.70 | 0.04 | 75.90 | 8.46 | 1824.32 | 54.75 |
| *Setaria viridis* | *Sporobolus* | 6.84 | 0.70 | 1.79 | 6.55 | 23.90 | 58.90 | 41.30 | 0.03 | 58.90 | 33.53 | 1774.55 | 89.59 |
| *Phyllostachys edulis* | *Phyllostachys* | 12.97 | 1.20 | 1.37 | 4.74 | 25.90 | 80.10 | 52.00 | 0.03 | 80.10 | 5.70 | 1825.53 | 56.20 |
| *Acer buergerianum* | *Acer* | 9.73 | 1.40 | 3.20 | 9.58 | 29.80 | 54.90 | 52.50 | 0.04 | 54.88 | 33.93 | 1808.46 | 81.34 |
| *Hylodesmum podocarpum* | *Sunhangia* | 13.59 | 0.50 | 4.23 | 3.69 | 36.60 | 75.30 | 57.80 | 0.02 | 75.30 | 10.59 | 1814.05 | 54.20 |
| *Solidago canadensis* | *Solidago* | 13.35 | 2.00 | 3.63 | 11.58 | 26.00 | 53.10 | 40.00 | 0.03 | 53.00 | 31.52 | 1844.19 | 101.15 |
| *Ophiopogon japonicus* | *Ophiopogon* | 8.63 | 1.30 | 4.96 | 9.10 | 5.49 | 60.88 | 32.78 | 0.20 | 55.19 | 28.99 | 1699.20 | 96.83 |
| *Pueraria montana* | *Pueraria* | 15.16 | 0.90 | 4.51 | 6.03 | 30.30 | 78.40 | 53.90 | 0.02 | 78.40 | 5.52 | 1833.20 | 55.66 |
| *Bidens pilosa* | *Bidens* | 15.56 | 2.10 | 3.41 | 13.61 | 33.80 | 67.90 | 53.10 | 0.04 | 67.90 | 14.40 | 1862.32 | 65.12 |
| *Vaccinium bracteatum* | *Vaccinium* | 9.29 | 1.60 | 3.53 | 14.27 | 32.30 | 67.00 | 49.00 | 0.04 | 67.00 | 22.07 | 1810.43 | 70.43 |
| *Euscaphis japonica* | *Euscaphis* | 12.31 | 2.00 | 3.08 | 13.92 | 25.80 | 68.50 | 45.70 | 0.04 | 68.40 | 17.15 | 1837.27 | 72.38 |
| *Vitis vinifera* | *Vitis* | 11.71 | 0.70 | 5.35 | 11.67 | 27.80 | 55.40 | 49.90 | 0.04 | 55.38 | 32.15 | 1805.64 | 84.00 |
| *Camellia oleifera* | *Camellia* | 8.09 | 1.10 | 2.33 | 10.02 | 14.42 | 62.10 | 41.40 | 0.03 | 41.34 | 28.68 | 1435.58 | 84.86 |
| *Spiraea cantoniensis* | *Spiraea* | 8.69 | 1.00 | 2.95 | 6.13 | 39.80 | 71.70 | 56.20 | 0.02 | 71.70 | 18.59 | 1793.45 | 58.54 |
| *Hedyotis diffusa* | *Scleromitrion* | 9.04 | 1.50 | 5.54 | 6.29 | 41.40 | 74.40 | 55.10 | 0.02 | 74.30 | 15.04 | 1805.20 | 57.49 |
| *Abelia chinensis* | *Abelia* | 8.21 | 1.50 | 3.26 | 13.92 | 24.90 | 65.60 | 41.00 | 0.02 | 65.50 | 24.67 | 1799.81 | 80.77 |
| *Lygodium flexuosum* | *Lygodium* | 11.74 | 1.50 | 2.76 | 24.12 | 32.90 | 64.30 | 57.10 | 0.05 | 64.21 | 22.41 | 1822.38 | 64.26 |

**Table S2** Main nutrients composition of diet plants for sika deer in winter

| **Species** | **Genus** | **CP** | **EE** | **ST** | **SS** | **ADL** | **NDF** | **ADF** | **Ash** | **TSC** | **TNC** | **GE** | **RFV** |
| --- | --- | --- | --- | --- | --- | --- | --- | --- | --- | --- | --- | --- | --- |
| *Rubus alceifolius* | *Rubus* | 10.68 | 1.70 | 3.55 | 10.04 | 21.36 | 60.46 | 43.70 | 0.13 | 38.97 | 27.03 | 1451.64 | 84.40 |
| *[Loropetalum chinense](http://www.iplant.cn/info/Loropetalum chinense)* | *Loropetalum* | 9.35 | 0.90 | 4.34 | 14.01 | 18.14 | 52.47 | 36.11 | 0.15 | 34.19 | 37.13 | 1479.59 | 107.73 |
| *Eurya japonica* | *Eurya* | 10.08 | 1.50 | 6.34 | 18.85 | 8.20 | 36.90 | 17.43 | 0.10 | 28.60 | 51.42 | 1669.91 | 189.86 |
| *Camellia japonica* | *Camellia* | 6.93 | 2.00 | 5.74 | 10.94 | 16.92 | 48.63 | 34.07 | 0.12 | 31.59 | 42.33 | 1510.45 | 119.30 |
| *Euonymus maackii* | *Euonymus* | 13.15 | 2.60 | 7.60 | 9.53 | 8.06 | 36.80 | 21.02 | 0.08 | 28.66 | 47.37 | 1717.60 | 183.32 |
| *Phyllostachys edulis* | *Phyllostachys* | 15.11 | 1.40 | 2.39 | 3.73 | 13.57 | 76.11 | 37.95 | 1.63 | 60.91 | 5.75 | 1555.84 | 72.53 |
| *Maclura tricuspidata* | *Maclura* | 16.56 | 1.90 | 4.48 | 7.01 | 16.38 | 59.34 | 34.12 | 0.13 | 42.83 | 22.07 | 1579.61 | 97.70 |
| *Cunninghamia lanceolata* | *Cunninghamia* | 10.93 | 1.70 | 4.20 | 8.13 | 22.09 | 65.14 | 54.25 | 1.85 | 41.20 | 20.38 | 1381.65 | 66.60 |
| *Rhododendron simsii* | *Rhododendron* | 8.49 | 2.80 | 5.58 | 15.84 | 22.33 | 52.18 | 42.18 | 0.09 | 29.77 | 36.44 | 1446.78 | 99.90 |
| *Celtis sinensis* | *Celtis* | 15.12 | 0.80 | 4.87 | 5.88 | 20.63 | 58.55 | 41.91 | 0.83 | 37.09 | 24.70 | 1448.81 | 89.37 |
| *Vaccinium bracteatum* | *Vaccinium* | 9.21 | 1.70 | 4.26 | 11.60 | 25.41 | 60.14 | 47.41 | 0.17 | 34.56 | 28.78 | 1371.23 | 80.39 |
| *Ilex cornuta* | *Ilex* | 8.22 | 3.70 | 4.99 | 11.47 | 16.73 | 58.06 | 38.51 | 0.10 | 41.23 | 29.92 | 1560.87 | 94.38 |
| *Sabia swinhoei* | *Sabia* | 8.74 | 2.10 | 4.41 | 15.17 | 22.14 | 46.49 | 38.93 | 0.13 | 24.22 | 42.53 | 1434.52 | 117.19 |
| *Cocculus orbiculatus* | *Cocculus* | 21.62 | 1.60 | 4.16 | 5.74 | 11.15 | 45.99 | 27.05 | 0.08 | 34.76 | 30.71 | 1697.21 | 137.18 |
| *Lysimachia christinae* | *Lysimachia* | 13.77 | 4.10 | 10.10 | 9.97 | 20.12 | 40.80 | 31.03 | 0.11 | 20.57 | 41.22 | 1547.26 | 147.57 |
| *Juniperus formosana* | *Juniperus* | 11.16 | 4.00 | 6.08 | 6.18 | 15.54 | 56.28 | 37.78 | 0.12 | 40.62 | 28.44 | 1606.38 | 98.29 |
| *Arrhenatherum elatius* | *Arrhenatherum* | 15.67 | 1.40 | 4.44 | 9.57 | 6.61 | 65.10 | 28.05 | 0.09 | 58.40 | 17.75 | 1731.58 | 95.81 |
| *Lindera aggregata* | *Lindera* | 8.38 | 2.90 | 4.85 | 13.91 | 30.54 | 69.23 | 53.30 | 0.57 | 38.12 | 18.92 | 1291.00 | 63.66 |
| *Rosa cymosa* | *Rosa* | 14.73 | 2.10 | 5.68 | 14.17 | 13.80 | 43.51 | 26.22 | 0.05 | 29.66 | 39.60 | 1619.26 | 146.40 |
| *Abelia chinensis* | *Abelia* | 14.21 | 1.70 | 4.74 | 12.76 | 18.32 | 52.04 | 34.37 | 0.06 | 33.66 | 31.99 | 1529.04 | 111.05 |
| *Broussonetia papyrifera* | *Broussonetia* | 10.15 | 1.90 | 5.35 | 1.68 | 4.55 | 34.30 | 19.65 | 0.15 | 29.60 | 53.50 | 1740.26 | 199.60 |
| *Pinus massoniana* | *Pinus* | 9.95 | 4.40 | 4.41 | 7.84 | 16.09 | 51.37 | 36.15 | 0.10 | 35.18 | 34.19 | 1598.72 | 109.99 |
| *Oxalis corniculata* | *Oxalis* | 19.04 | 2.00 | 5.96 | 11.44 | 14.38 | 41.19 | 25.91 | 0.11 | 26.71 | 37.66 | 1633.00 | 155.18 |

**Table S3** Evaluation table of gray correlation analysis of food plants for sika deer in summer and winter

| **Plant taxa** | **Summer diet plants** | **Genus** | **Score** | **Ranking** | **Plant taxa** | **Winter diet plants** | **Genus** | **Score** | **Ranking** |
| --- | --- | --- | --- | --- | --- | --- | --- | --- | --- |
| Arbor | *Pistacia chinensis* | *Pistacia* | 0.69 | 1 | Shrub | *Eurya japonica* | *Eurya* | 0.66 | 1 |
| Herbs | *Ophiopogon japonicus* | *Ophiopogon* | 0.63 | 2 | Herbs | *Lysimachia christinae* | *Lysimachia* | 0.65 | 2 |
| Herbs | *Solidago canadensis* | *Solidago* | 0.59 | 3 | Arbor | *Euonymus maackii* | *Euonymus* | 0.62 | 3 |
| Vine | *Vitis vinifera* | *Vitis* | 0.58 | 4 | Herbs | *Arrhenatherum elatius* | *Arrhenatherum* | 0.62 | 4 |
| Herbs | *Viola philippica* | *Viola* | 0.58 | 5 | Shrub | *Broussonetia papyrifera* | *Broussonetia* | 0.61 | 5 |
| Vine | *Pueraria montana* | *Pueraria* | 0.57 | 6 | Vine | *Cocculus orbiculatus* | *Cocculus* | 0.59 | 6 |
| Arbor | *Cunninghamia lanceolata* | *Cunninghamia* | 0.57 | 7 | Arbor | *Pinus massoniana* | *Pinus* | 0.59 | 7 |
| Herbs | *Bidens pilosa* | *Bidens* | 0.57 | 8 | Shrub | *Rosa cymosa* | *Rosa* | 0.58 | 8 |
| Vine | *Lygodium flexuosum* | *Lygodium* | 0.56 | 9 | Herbs | *Oxalis corniculata* | *Oxalis* | 0.58 | 9 |
| Shrub | *Euscaphis japonica* | *Euscaphis* | 0.56 | 10 | Arbor | *Juniperus formosana* | *Juniperus* | 0.57 | 10 |
| Shrub | *Abelia chinensis* | *Abelia* | 0.55 | 11 | Shrub | *Ilex cornuta* | *Ilex* | 0.56 | 11 |
| Herbs | *Hedyotis diffusa* | *Scleromitrion* | 0.55 | 12 | Shrub | *Rhododendron simsii* | *Rhododendron* | 0.56 | 12 |
| Shrub | *Rubus alceifolius* | *Rubus* | 0.55 | 13 | Shrub | *Abelia chinensis* | *Abelia* | 0.54 | 13 |
| Herbs | *Phyllanthus urinaria* | *Phyllanthus* | 0.54 | 14 | Vine | *Sabia swinhoei* | *Sabia* | 0.54 | 14 |
| Herbs | *Phyllostachys edulis* | *Phyllostachys* | 0.54 | 15 | Arbor | *Camellia japonica* | *Camellia* | 0.53 | 15 |
| Arbor | *Acer buergerianum* | *Acer* | 0.53 | 16 | Arbor | *Maclura tricuspidata* | *Maclura* | 0.53 | 16 |
| Herbs | *Setaria viridis* | *Sporobolus* | 0.52 | 17 | Shrub | *[Loropetalum chinense](http://www.iplant.cn/info/Loropetalum chinense)* | *Loropetalum* | 0.52 | 17 |
| Shrub | *Vaccinium bracteatum* | *Vaccinium* | 0.52 | 18 | Shrub | *Rubus alceifolius* | *Rubus* | 0.50 | 18 |
| Arbor | *Camellia oleifera* | *Camellia* | 0.52 | 19 | Shrub | *Vaccinium bracteatum* | *Vaccinium* | 0.49 | 19 |
| Herbs | *Hylodesmum podocarpum* | *Sunhangia* | 0.51 | 20 | Herbs | *Phyllostachys edulis* | *Phyllostachys* | 0.49 | 20 |
| Herbs | *Oxalis corniculata* | *Oxalis* | 0.51 | 21 | Arbor | *Lindera aggregata* | *Lindera* | 0.48 | 21 |
| Arbor | *Quercus fabri* | *Quercus* | 0.49 | 22 | Arbor | *Celtis sinensis* | *Celtis* | 0.45 | 22 |
| Vine | *Smilax china* | *Smilax* | 0.49 | 23 | Arbor | *Cunninghamia lanceolata* | *Cunninghamia* | 0.42 | 23 |
| Shrub | *[Loropetalum chinense](http://www.iplant.cn/info/Loropetalum chinense)* | *Loropetalum* | 0.49 | 24 |  |  |  |  |  |
| Shrub | *Spiraea cantoniensis* | *Spiraea* | 0.48 | 25 |  |  |  |  |  |
| Shrub | *Rhododendron simsii* | *Rhododendron* | 0.46 | 26 |  |  |  |  |  |
| Herbs | *Lespedeza pilosa* | *Lespedeza* | 0.46 | 27 |  |  |  |  |  |

**Table S4** Topological properties of the SPIEC-EASI co-occurrence networks under SLu and WLu

| Network indices | SLu | WLu |
| --- | --- | --- |
| Total nodes | 154 | 90 |
| Total links | 2025 | 854 |
| Positive links (%) | 79.41 | 20.59 |
| Negative links (%) | 64.64 | 35.36 |
| Average Degree | 26.30 | 18.98 |
| Network Diameter | 7 | 5 |
| Density | 0.172 | 0.213 |
| Modularity | 0.21 | 0.24 |
| Average path length | 2.17 | 2.13 |
| Average clustering coefficient | 0.643 | 0.636 |
| Proportion (Positive/total) | 65.26 | 54.28 |
